# Evaluation of Volumetric Change of Intracerebral Hemorrhage in Patients Treated with Thrombolysis for Intraventricular Hemorrhage

**DOI:** 10.1007/s12028-020-01054-7

**Published:** 2020-07-31

**Authors:** Franziska Staub-Bartelt, Jasper Hans van Lieshout, Thomas Beez, Rainer Kram, Daniel Hänggi, Kerim Beseoglu

**Affiliations:** 1 Department of Neurosurgery, Medical Faculty, Heinrich-Heine University Düsseldorf, Moorenstraße 5, 40225 Düsseldorf, Germany; 2 Department of Anesthesiology, Medical Faculty, Heinrich-Heine University Düsseldorf, Düsseldorf, Germany

**Keywords:** Intraventricular hemorrhage, Intracerebral hematoma, Intrathecal lysis, Volumetric change

## Abstract

**Background:**

Intraventricular hemorrhage (IVH) is often caused by irruption of intracerebral hemorrhage (ICH) of basal ganglia or thalamus into the ventricular system. Instillation of recombinant tissue plasminogen activator (rtPA) via an external ventricular drainage (EVD) has been shown to effectively decrease IVH volumes while the impact of rtPA instillation on ICH volumes remains unclear. In this series, we analyzed volumetric changes of ICH in patients with and without intrathecal lysis therapy.

**Methods:**

Between 01/2013 and 01/2019, 36 patients with IVH caused by hemorrhage of basal ganglia, thalamus or brain stem were treated with rtPA via an EVD (Group A). Initial volumes were determined in the first available computed tomography (CT) scan, final volumes in the last CT scan before discharge. During the same period, 41 patients with ICH without relevant IVH were treated without intrathecal lysis therapy at our neurocritical care unit (Group B). Serial CT scans were evaluated separately for changes in ICH volumes for both cohorts using OsiriX DICOM viewer. The Wilcoxon signed-rank test was performed for statistical analysis in not normally distributed variables.

**Results:**

Median initial volume of ICH for treatment Group A was 6.5 ml and was reduced to 5.0 ml after first instillation of rtPA (*p *< 0.01). Twenty-six patients received a second treatment with rtPA (ICH volume reduction 4.5 to 3.3 ml, *p *< 0.01) and of this cohort further 16 patients underwent a third treatment (ICH volume reduction 3.0 ml to 1.5 ml, *p *< 0.01). Comparison of first and last CT scan in Group A confirmed an overall median percentage reduction of 91.7% (*n *= 36, *p* < 0.01) of ICH volumes and hematoma resolution in Group A was significantly more effective compared to non-rtPA group, Group B (percentage reduction = 68%) independent of initial hematoma volume in the regression analysis (*p *= 0.07, mean 11.1, 95%CI 7.7–14.5). There were no adverse events in Group A related to rtPA instillation.

**Conclusion:**

Intrathecal lysis therapy leads to a significant reduction in the intraparenchymal hematoma volume with faster clot resolution compared to the spontaneous hematoma resorption. Furthermore, intrathecal rtPA application had no adverse effect on ICH volume.

**Electronic supplementary material:**

The online version of this article (10.1007/s12028-020-01054-7) contains supplementary material, which is available to authorized users.

## Introduction

Spontaneous intracerebral hemorrhage (ICH) is a life-threatening event leading to high mortality rates and permanent disability in surviving patients [1]. Intraventricular hemorrhage (IVH)—an extension of the ICH due to irruption of the bleeding into the ventricular system—is reported in more than 50% of patients with ICH and its volume is among the main predictors for poor outcome [2–5]. Due to its high impact on mortality and morbidity, diverse treatment strategies for IVH have been evaluated in the past. However, a clinical benefit from reduction in IVH due to irrigation of fibrinolytic substances through an external ventricular drainage (EVD) has been difficult to demonstrate in humans. Studies provide evidence for a reduction in all-cause mortality but shows no improvement in functional outcome [6–9].

Intraparenchymal hematoma expansion following intrathecal lysis therapy of IVH could explain these disappointing results, since neurological impairment due to ICH is directly associated with the clot size [10]. The aim of our study was to determine the effect of intrathecal lysis therapy on the volume of ICH. We here report our findings in 36 patients with ICH and concomitant IVH treated with intrathecal fibrinolysis and compared ICH volumes and occurrence of adverse events with a group of patients with ICH either with or without IVH treated with EVD alone.

## Methods

We performed a retrospective analysis of a single-center cohort to investigate the effect of intrathecal recombinant tissue plasminogen activator (rtPA) on the volumetric changes of ICH associated with intrathecal lysis therapy. The study was approved by the local ethical committee (Study number: 2018-295). Reporting of this study was according to the strengthening the reporting of observational studies in epidemiology (STROBE) guidelines for observational studies (Supplementary Material) [11].

### Patients

**Fig. 1:**
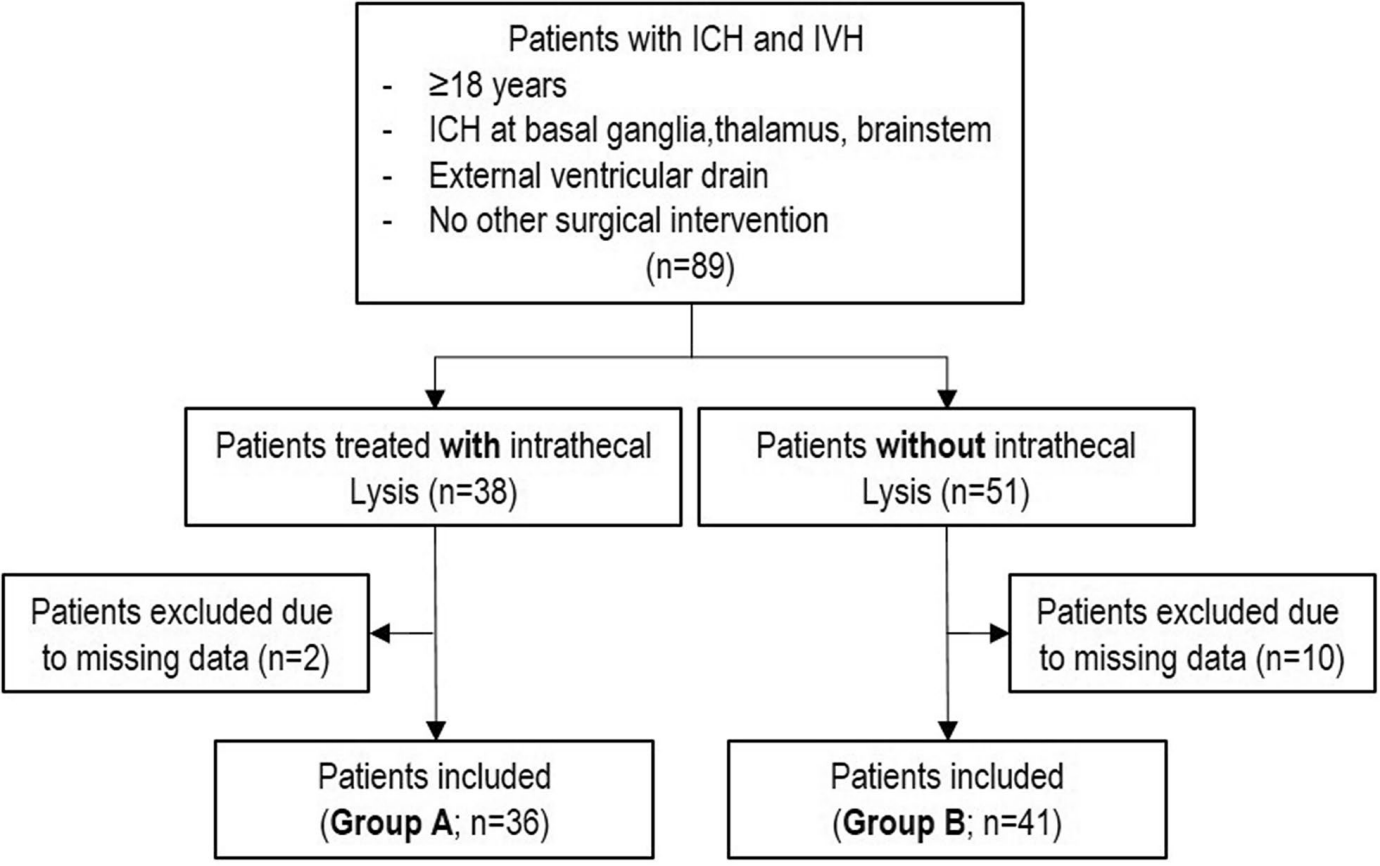
Flowchart illustrating patient screening and selection. Patients were screened regarding general criteria (age, localization of ICH, EVD, no other surgical intervention) and then divided into two groups. Group A patients receiving intrathecal lysis, Group B patients without intrathecal lysis. Afterwards, patients with missing data (e.g., no follow-UP CT scans) in both cohorts were excluded

We identified all consecutive patients admitted to our neurocritical care unit at the University Hospital Düsseldorf with an IVH due to ICH and treated with rtPA via an EVD, between January 2013 and January 2019. Inclusion criteria were: (1) age 18 years and older, (2) origin of hemorrhage located either at the basal ganglia, thalamus or brain stem with relevant extension into any ventricle, (3) availability of initial and follow-up computed tomography (CT) scans and (4) at least one documented treatment with intrathecal lysis (Fig. 1). A relevant IVH was defined as a blood volume impending to cause a hydrocephalus as evaluated by the treating physician with obstruction of the Foramina Monroi and/or the aqueduct.

For definition of a control group (Group B), we furthermore screened the patient database for patients with (1) age 18 years and older, (2) ICH located at either basal ganglia, thalamus or brain stem, (3) receiving an EVD without an indication for intrathecal lysis (e.g., no or insignificant amounts of IVH) treated during the same period of time.

Patients who underwent surgery for evacuation of the intracerebral hematoma, patients with lobar ICH and ICH related to aneurysm rupture, arteriovenous malformation rupture or neoplasm as well as traumatic hemorrhages were excluded.

Indication for EVD insertion in patients with ICH with or without IVH (Group A and Group B) were unconsciousness with indication for intubation, intubation due to other reasons (e.g., pulmonary insufficiency) and was established to monitor ICPs during the sedation/acute unconscious phase where patients could not undergo adequate neurological assessments. Additionally, for patients with ICH and IVH (Group A) indication for intrathecal lysis required EVD insertion. Indication for intrathecal lysis were: hydrocephalus/neurological deterioration due to tri-or tetra ventricular blood collection, particularly with localization in the III and IV ventricle.

All patients included in Group A were treated with rtPA (Actilyse^®^, Boehringer-Ingelheim, Germany) within 12 h after initial CT scan. For each application, 2.5 mg rtPA was dissolved in 2 ml of saline solution. Actilyse was instilled directly into the ventricle followed by instillation of 2 ml saline solution in order to guarantee intrathecal application. The EVD was then closed for 30–45 min, depending on individual patient tolerance. Upon reopening of the EVD, the drainage system was kept at a reduced pressure threshold (10–12 mmHg) to achieve sufficient clearance of blood. This procedure was repeated every 12 h until a sufficient reduction in intraventricular blood in the third and/or fourth ventricle was achieved. We performed repeat CT imaging every 24 h to determine the treatment effect as reported in previous publications [12, 13].

### Outcome

**Fig. 2:**
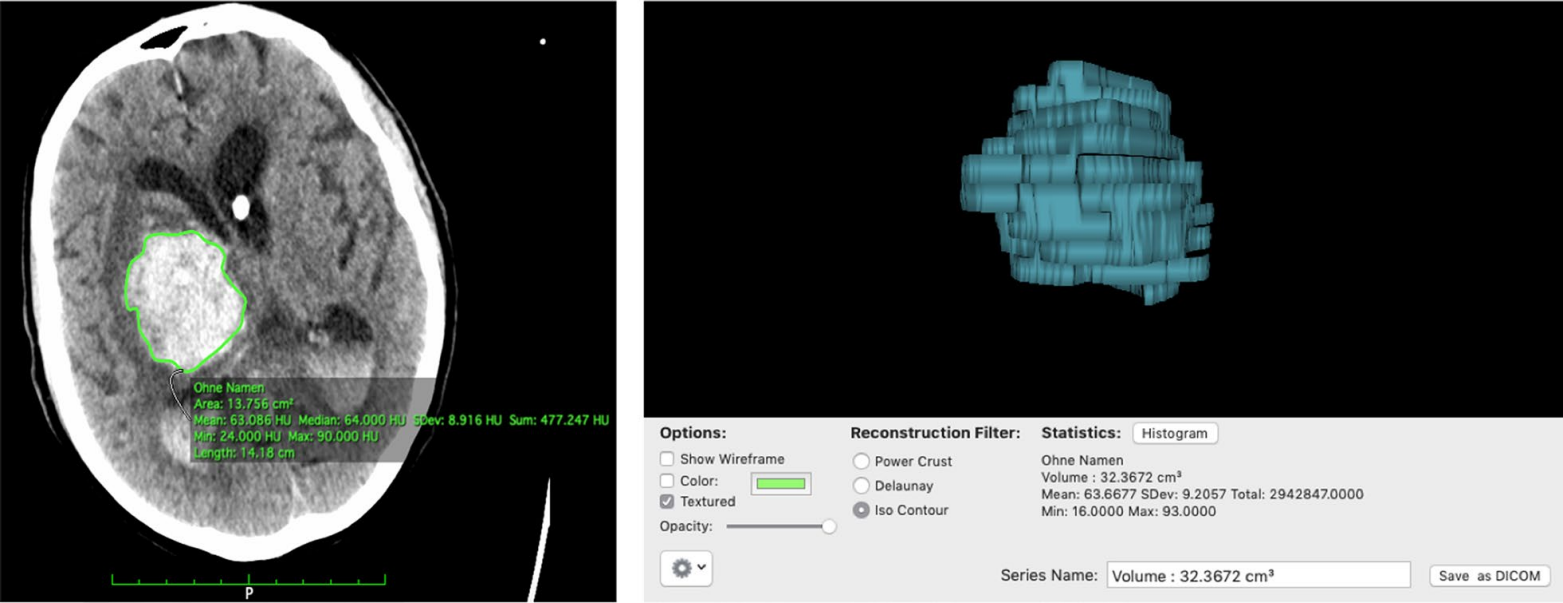
Example for the region of interest (ROI) volumetry using OsirixLite (Pixmeo SARL, Switzerland). Axial CT scans were checked for slides showing hyperdensity relating to ICH, and hematoma outlines were marked on corresponding slides (left). Hematoma volume was then calculated automatically using ROI volumetry function of the software (right)

Primary outcome measure was the median volume (ml) of ICH on repeat CT imaging. Hematoma volume for every patient at every defined point of time was measured by one of the authors (FSB) using Region of Interest (ROI) volumetry in OsirixLite (Pixmeo SARL, Switzerland). The hematoma outline, defined as hyperdensity compared to brain parenchyma, was outlined manually on axial CT slices with 2–5 mm thickness. Hematoma volume was then calculated automatically using ROI volumetry function of the software (Fig. 2).

The first available CT scan showing ICH/IVH was defined as “initial” scan irrespective whether further CT scans without any intervention existed or not. First follow-up CT after first instillation of rtPA was defined as “first,” second available CT scan after repeated instillation was defined as “second,“ third CT scan after further instillation of rtPA was defined as “third.“ Accordingly last available CT scan before discharge of the patients was defined as “final.“ CT scans were generally done within 24 h after rtPA application. The number of CT scans were not representative for the number of intrathecal lysis administration. For the 41 patients from Group B only analyses of volume for the time periods “initial” and “final” were conducted.

### Statistical Analysis

Categorical data are presented as counts and percentages and continuous variables as means with standard error of the mean (SEM) or medians with interquartile ranges (IQRs), depending on the normality of the data.

For the primary outcome measure, we calculated median and interquartile ranges (IQR) instead of mean values and standard deviation in order to avoid distortion by outliers and extremities due to the small sample size. The Shapiro–Wilk test was used to test for normal distribution. Hereafter, non-parametric testing was used for related samples to determine changes in hematoma volumes over the treatment period (Wilcoxon signed-rank test). Group differences between Group A and B were evaluated using Mann–Whitney *U* test. Additionally, we performed a regression analysis with hematoma volume reduction (in %) as dependent variable and rtPA application and initial hematoma volume as contributing independent variables. The Type I error was set at 0.05 and the tests were 2-tailed. Statistical analyses were performed using IBM SPSS Statistics Version 26 (IBM Corporation, USA).

## Results

We included 36 patients with IVH due to ICH treated with rtPA via an EVD (Group A, Supplement Material Table 1) and 41 patients with ICH with or without IVH without intrathecal rtPA application (Group B, Supplement Material Table 2). All epidemiological data including main risk factors for ICH are summarized in Table 1. Median initial ICH volume in cohort A (*n *= 36) was 6.5 ml (IQR 5.3–12.6 ml) and was reduced to 5.0 ml (IQR 3.0–9.9 ml) after first treatment with intrathecal rtPA (*p *< 0.01).

**Table 1:** Details and comparison of patient Group A and B

	Intrathecal lysis Group A	No intrathecal lysis Group B	*p*
*N*	36	41	
Female			
Absolute (%)	23 (64%)	14 (34%)	**< 0.01**
Age			
Median (IQR)	67 (59–74)	63 (52–70)	0.32
Location absolute (%)			
Basal Ganglia	26 (72%)	30 (73%)	0.93
Thalamus or Brainstem	10 (28%)	11 (27%)	
*Clot volume on CT scan (ml)*			
Median (IQR)			
Initial	6.5 (5.3–12.6)	8.9 (4.3–13.1)	0.74
After first lysis	5.0 (3.0–9.9)	N/A	
After second lysis	3.3 (1.6–8.4)	N/A	
After third lysis	1.5 (0.7–6.5)	N/A	
Final	0.5 (0–3.9)	3.1 (0.6–7.1)	**0.02**
Reduction in clot volume in percent initial to final CT scan			
Mean (95% confidence interval)	81.8 (73.5–90.2)	63.4 (53.4–73.4)	**< 0.01**
Time initial CT scan—1st lysis (h)			
Median (IQR)	10.0 (6.8–14.0)	N/A	
Days (initial–final CT scan)			
Median (IQR)	10 (7–14)	11 (9–17)	0.20
*Risk factors for IVH*			
Absolute (%)			
Arterial hypertension	33 (92%)	32 (78%)	0.10
Diabetes	4 (11%)	6 (15%)	0.65
Coronary artery disease	7 (19%)	3 (7%)	0.12
Alcohol	5 (14%)	3 (7%)	0.35
Nicotine	11 (31%)	5 (12%)	**0.05**
Antiplatelet	7 (19%)	4 (10%)	0.23
Anticoagulation	6 (17%)	4 (10%)	0.37
EVD-related infections (%)	0	0	–
CSF shunt rate (%)	20 (55%)	14 (34%)	0.24
Timing of permanent shunting in days			
Median (IQR)	9.5 (7–12)	11 (9–12)	0.26

Bold values represent significant values (*p* < 0.05)

Details of all patients from Group A (intrathecal lysis) and Group B (no intrathecal lysis). Location thalamus and brainstem have been grouped due to the small group size. IQR interquartile range, CT scan computed tomography scan

**Fig. 3:**
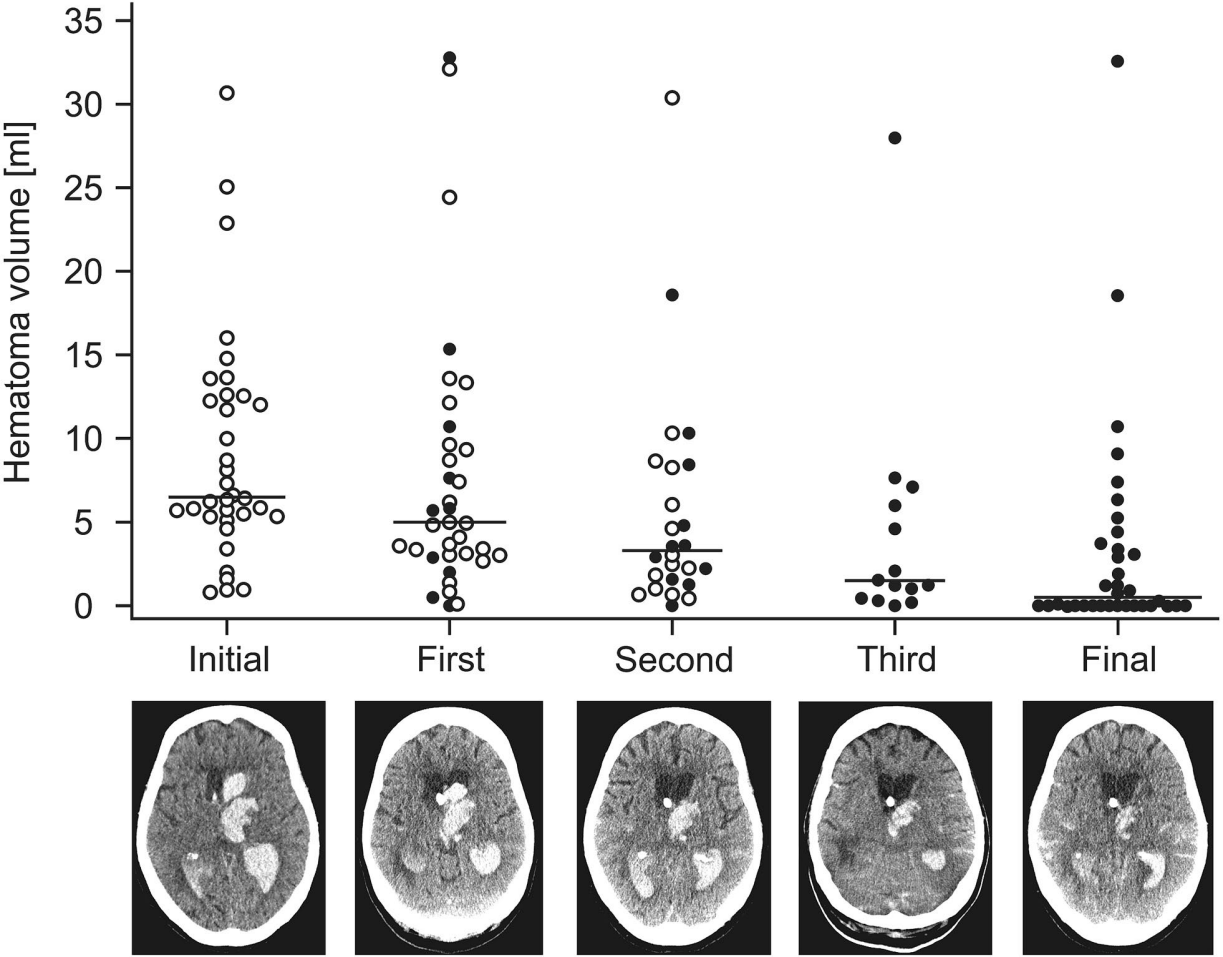
Visualization of hematoma volumes in Group A. Hematoma volume (ml) in the initial CT scan and after first, second and third application of recombinant tissue plasminogen activator (rtPA) as well as in the final CT scan before discharge. Circles signify patients receiving a repeated rtPA dose after CT scan, dots signify patients who did not receive further rtPA application. Patient #14 from Group A was omitted in this figure for illustrative reasons due to a very high hematoma volume; however, the value was included when calculating the medians

26 out of 36 patients received a second instillation of rtPA. Follow-up CT scan showed a decrease in median hematoma volume for this subgroup [4.5 ml (IQR 3.1–9.5 ml) to 3.3 ml (IQR 1.6–8.4 ml); *p *< 0.01]. A further 16 out of 26 patients received a third intrathecal application of rtPA, which further decreased the mean hematoma volume from 3.0 ml (IQR 1.4–8.5 ml) to 1.5 ml (IQR 0.7–6.5 ml); (*p *< 0.01, Fig. 3).

**Fig. 4:**
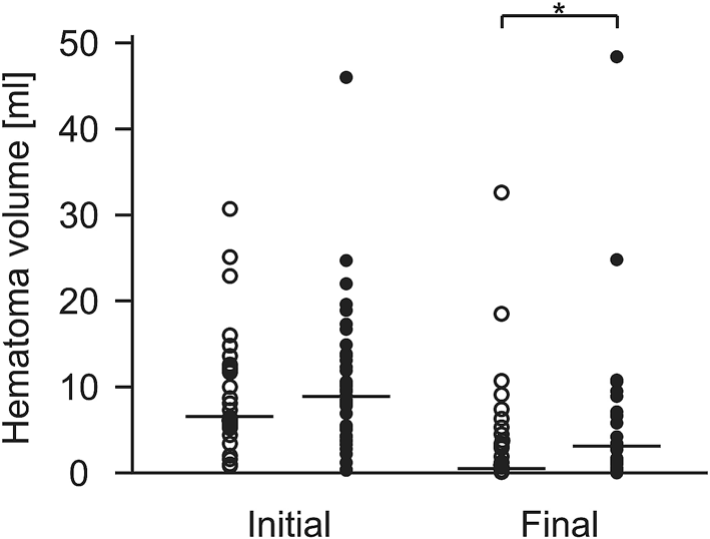
Comparison of initial and final hematoma volume in Group A and B. Initial and final hematoma volume for patients receiving intrathecal lysis therapy (Group A, circles) compared to patients without intrathecal lysis therapy (Group B, dots). Comparison of hematoma volume in the final CT scan demonstrated a significant difference between both groups (*p *< 0.02). Patient #14 from Group A was omitted in this figure for illustrative reasons due to a very high hematoma volume; however, the value was included when calculating the medians

Comparison of initial and final hematoma volume in Group A showed an absolute median decrease from 6.5 ml (IQR 5.3–12.6 ml) to 0.5 ml (IQR 0.0–3.9 ml). In Group B, median hematoma volume decreased from initially 8.9 ml (IQR4.3–13.1 ml) to 3.1 ml (IQR 0.6–7.1 ml). Patients from Group A showed a significantly higher clot resolution rate as compared to Group B (*p *< 0.02, Fig. 4). In a regression analysis with hematoma volume reduction in percent (mean 72.1%; 95%CI 65.3–78.8%) as the dependent variable and initial hematoma volume and intraventricular rtPA application as independent variables only rtPA application (proportion 0.46; 95%CI 0.35–0.57) contributed significantly (*p *< 0.01) to the model. Initial hematoma volume (mean 11.1 ml, 95%CI 7.7–14.5 ml) was excluded (*p *= 0.07).

Median time between initial CT and first administration of rtPA was 10.0 h (IQR 6.8–14.0 h).

34 patients received repeated CT scans every 24 h after instillation of either one or two doses of rtPA per day. In 2 patients (subject 1 and 2), the interval between initial CT scan, start of lysis and first control CT was doubled to 48 h. Both patients’ treatment were finalized after the first CT scan. The median time period between the initial and final CT scan for Group A was 10.0 days (IQR 7.0–14.0). The median period between initial and final CT scan for Group B was 11 days (IQR 9.0–17.0).

No patient experienced an increase in hematoma volume after application of rtPA. However, 6 patients showed a hematoma progression between initial CT scan and start of intrathecal lysis (Table 2). All of these patients were treated with rtPA after hematoma progression and showed a significant hematoma reduction by the time of discharge (***p *= **0.03). Furthermore, 3 patients of Group B showed higher hematoma volume at final CT scan compared to initial CT scan (subject 10, 21, 34, for detailed volumes please refer to Table 2 of supplement material). As these patients received no intervention, hematoma growth was due to natural progression and not related to any intervention.

**Table 2:** Patients from cohort A with a spontaneous hematoma expansion before initiation of intrathecal rtPA (*n *= 6)

Patient	Location	CT scan admission	CT scan pre-lysis	1st CT scan post-lysis	CT scan discharge
		Volume (ml)	Volume (ml)	Volume (ml)	Volume (ml)
8	Basal ganglia	22.9	33.1	24.4	3.4
11	Basal ganglia	5.9	23.3	15.3	9.1
13	Basal ganglia	30.7	34.0	32.1	18.5
18	Basal ganglia	0.1	3.8	2.7	0.0
28	Basal ganglia	25.1	33.0	32.8	32.6
29	Basal ganglia	5.3	5.9	5.7	3.7
		14.4 [5.3–25.1]	28.1 [10.3–33.1]	19.8 [5.7–32.1]	6.4 [3.4–18.5]

A subgroup of patients in Group A showing details of hematoma progression between initial CT scan and last CT before intrathecal lysis. As not all patients received repeated CT scans before treatment, CTs on admission were used for calculation of initial hematoma volume. Group values are stated as median values with interquartile ranges

Overall, we did not see any infection related to intrathecal lysis therapy. Permanent shunting was performed in 55% of patients in Group A and 34% of patients in Group B during hospitalization. Median time from initial CT to permanent shunting for Group A was 9.5 days (IQR 7–12) and for Group B 11 days (IQR 9–12) (Table 1).

## Discussion

Our analysis reveals three relevant aspects. First, intraventricular application of rtPA in patients with ICH and concomitant IVH significantly decreases parenchymal hematoma volume without direct application into the hematoma. Second, rtPA application accelerates intraparenchymatous hematoma reduction compared to rtPA-untreated patients. Third, the intrathecal application of rtPA does not increase the risk of parenchymal hematoma expansion or hemorrhagic complications.

The efficacy of intraventricular clot resolution by application of rtPA via an EVD has been demonstrated before [13]; however, the focus was placed on IVH and to our knowledge data on the effect on the intraparenchymatous hematoma has not been published before.

Direct application of fibrinolytic agents into an intracerebral hematoma via image-guided catheter placement significantly reduces clot volume but investigated patients had no IVH and required surgery to place the catheter [10]. In patients with deep-seated hematomas extending into the ventricles, insertion of an EVD is necessary in most cases to treat concomitant hydrocephalus and allow for ICP monitoring. This simultaneously provides clinicians with a route for application of rtPA without the need for an additional intervention. Our analysis demonstrates that the intraventricular rtPa application leads to significant reduction in ICH volume. It appears likely that rtPA diffuses into the parenchyma via the breach in the ventricle wall and the local effects are comparable to direct application of fibrinolytic agents into the intraparenchymal hematoma without the necessity of an additional catheter [10]. However, the underlying biophysiological processes remain obscure in detail.

Compared to the spontaneous hematoma resorption rate, treated patients showed a significantly greater reduction in ICH hematoma volume over time.

Comparable to other reports, we did not experience rebleeding complications attributable to rtPA application. In the MISTIE III trial, no significant difference in the prevalence of adverse events like symptomatic bleeding between the standard medical care and interventional group was observed, even if rtPA was instilled directly into the residual hematoma after surgical clot removal [10]. Similarly, the CLEAR III trial showed a very low risk for new bleeding events under rtPA instillation therapy. It was reported that 2.4% (*n *= 6) of the patients suffered from symptomatic hemorrhages, only three of them (1.2%) during the dosing phase [14]. Hematoma expansion in 6 patients from the treated cohort occurred before intrathecal therapy and was thus unrelated to rtPA application and in line with previously reported hematoma progression during the first 24 h after hemorrhage [15].

Study limitations include the retrospective design and particularly the small number of patients in both cohorts. Regarding the retrospective character of the study, we would like to underline the following limitation. The relevance of faster ICH clot resolution for neurological outcome was not evaluated in the present data analysis. This was due to missing neurological outcome parameters in a large number of patients which excluded possibilities for any reliable assessment. Still, the procedure described was safe with regard to hematoma progression and seems to have no negative consequences on neurological outcome, but we cannot provide evidence for the treatment protocol with regard to improved functional outcome.

In addition to the small sample size, the median ICH size was considerably smaller compared to larger cohort studies as the MISTIE III trial. In our analysis, we focused on the effect of intrathecal lysis on ICH volumes; therefore, patients with larger ICH clot sizes with indication for any surgical intervention (e.g., minimal invasive treatments, evacuation of ICH) addressing the ICH were excluded as hematoma volumes would not have been comparable regarding the specific aim of the present study.

At last, limitations of CT evaluation need to be discussed. First, hematoma evaluation was not blinded to the groups. Secondly, the software that was used (Osirix Lite) is a free software version of Osirix MD, a widely used medical image viewer enabling various tools for processing of MRI/CT scans and other medical imaging. The full version is FDA cleared and CE IIa labelled and might be used for radiological diagnosis. We decided to use Osirix Lite as this is a freely available high-quality software version even though some tools are missing compared to Osirix MD. For CT evaluation, ROI volumetry was needed and still available through Osirix Lite. In order to verify the results, we randomly evaluated hematoma volumes by ABC/2 score and could not find any major discrepancy. A last potential confounder might be the decrease in hematoma density over time due to degradation of hemoglobin. Approximately 3–20 days after acute onset of ICH, the appearance of intraparenchymal hematoma in CT scans becomes less intense [16, 17]. The median time between initial CT and initiation of therapy in the intervention Group A was 10 h followed by repetitive CT scans every 24 h (except in 2 subjects). Thus, the treatment group received the majority of CT scans in the hyperacute and acute ICH stages, where the clot appears to be clearly hyperdense allowing a clear identification of parenchyma clot borders in the ROI volumetry. For comparison of initial and last CT in both cohorts, the decrease in hematoma density might have had an influence on evaluation.

According to its aim and study design, this study confirmed feasibility, safety and efficacy in clot resolution and our findings provide a basis for further prospective data collections. A controlled prospective study would be required to further elucidate, if the effect demonstrated is transferable to larger patient cohorts.

## Conclusion

We could demonstrate that intraventricular rtPA application significantly reduces ICH volumes in patients with IVH secondary to ICH in a small patient cohort. Furthermore, intrathecal thrombolysis leads to accelerated reduction in ICH volume compared to patients treated with EVD alone. The risk of procedure-related complications was not increased, especially with regard to expansion of intraparenchymatous hematoma. Nevertheless, a prospective randomized trial will be mandatory to define new treatment strategies.

## Electronic supplementary material

Below is the link to the electronic supplementary material.

Supplementary material 1 (DOCX 87 kb)

## Abbreviations

IVH: Intraventricular hemorrhage
ICH: Intracerebral hemorrhage
rtPA: Recombinant tissue plasminogen activator
EVD: External ventricular drainage
STROBE: Strengthening the Reporting of Observational Studies in Epidemiology
CT: Computed tomography

## Availability of Data and Materials

All data generated or analyzed during this study are included in this published article and its supplementary information files.
